# Clostridium sordellii: A Cause of Toxic Shock Syndrome After a Breach in the GI Tract

**DOI:** 10.7759/cureus.44604

**Published:** 2023-09-03

**Authors:** Sydney Pence, Rajshri Joshi, Faris Shweikeh, Mohamad Mouchli, Kasturi Shrestha

**Affiliations:** 1 Internal Medicine, Ohio University Heritage College of Osteopathic Medicine, Warrensville Heights, USA; 2 Internal Medicine, Cleveland Clinic Akron General, Akron, USA; 3 Gastroenterology and Hepatology, Cleveland Clinic Foundation, Cleveland, USA; 4 Infectious Disease, Cleveland Clinic Akron General, Akron, USA

**Keywords:** colonic fistula, bacterial translocation, gastrointestinal, septic shock [ss], clostridium sordelli

## Abstract

*Clostridium sordellii* is a highly virulent microorganism that causes serious infections, most commonly of the uterus and perineum. It has a high associated mortality rate due to the various toxins that it produces. A review of the literature suggests that knowledge surrounding its proper management is limited. This report describes a case of *Clostridium sordellii* causing toxic shock syndrome posttranslocation through the GI tract. A 69-year-old man with a past medical history of renal cell carcinoma and small bowl obstruction complicating transverse colostomy presented to the emergency room with back pain and rigors. Vital signs showed that he was in hemodynamic shock, and imaging revealed a left renal mass invading the adjacent splenic flexure of the colon. There was also a significant leukemoid reaction. After receiving a series of antibiotics, blood cultures revealed *Clostridium sordellii* as the pathogen of interest. As the first report of its kind, we identify a unique presentation of this organism, serving as a primary example of a different setting that clinicians should be aware of while at the same time highlighting a successful course of therapy for this often deadly organism.

## Introduction

*Clostridium sordellii*, first identified in 1922 by Alfredo Sordelli, is a beta-hemolytic anaerobic gram-positive spore-forming rod [1]. It is typically found in the soil and guts of many animals, including humans [1]. When found pathologically in humans, *C. sordellii* is almost exclusively reported with infections of the uterus and perineum; however, there have been rare cases of infection in other locations of the body reported post-operatively or with intravenous drug use [1-4]. In most of the reported cases of clostridial bacteremia, patients were post-surgical, immunocompromised, or had a malignancy [1]. Suppressed immune systems in many have also been a cause of delayed presentation of signs of infection, thus making the organism invariably fatal [1].

Unfortunately, *C. sordellii* is highly virulent, causing death in nearly 70% of cases [1,5]. Its virulence is achieved with exotoxins, primarily the lethal and hemorrhagic toxins [1]. Infection with *C. sordellii* typically causes an acute-onset leukemoid reaction accompanied by hypotension and tachycardia [1]. Some reports have demonstrated this pathogen to cause a capillary leak syndrome, leading to hemoconcentration [1]. Even more severely, there have been reports of *C. sordellii* almost exclusively involving the uterus or perineum, causing toxic shock syndrome [2,6]. This presentation is unlike most presentations of postoperative sepsis, which is typically caused by *Staphylococcus aureus* and begins as a surgical site infection [7,8].

Little guidance exists regarding the treatment of *C. sordellii*. Some older studies suggest that the infection is responsive to beta-lactams, clindamycin, tetracycline, and chloramphenicol [9], while newer studies identify resistance patterns to tetracyclines [10]. This report presents a recent case that highlights the diagnosis and treatment of *Clostridium sordellii* causing toxic shock syndrome in the setting of a hemorrhagic necrotic renal mass and its fistulization with the adjacent splenic flexure of the colon. As the first report of its kind, this report identifies a unique presentation of *C. sordellii* for clinicians to be aware of and demonstrates a successful course of therapy.

## Case presentation

**Figure 1:**
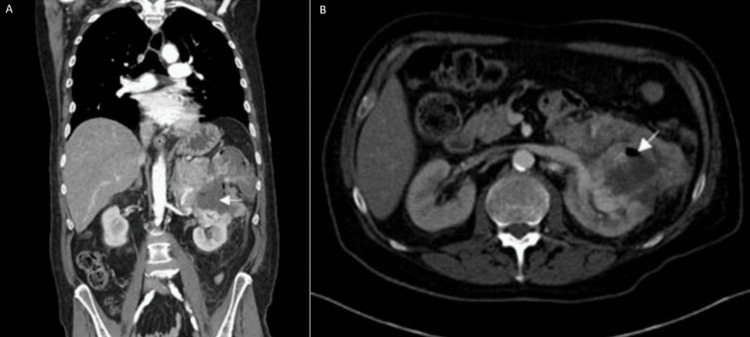
Radiographic images of a 69-year-old male with a history of renal cell carcinoma presenting acutely with back pain and rigors. Abdominal CT scan in A) coronal view and B) axial view. Arrows point to the area of the necrotic renal mass invading the colon.

A 69-year-old man presented to the emergency room with back pain and uncontrollable shaking. His past medical history is significant for metastatic renal cell carcinoma, a penicillin allergy, and a surgical history of small bowel obstruction two weeks after undergoing a transverse colostomy and a one-week post-renal biopsy. On a physical exam, the patient was confused and found to be tachycardic, tachypneic, hypotensive, and febrile. Pertinent labs included a lactate of 8mmol/L (0.5-2.2 mmol/L), an initial hemoglobin of 10g/dL that fell to 7.7g/dl (11.5 - 15.5 g/dL), and a WBC count of 16,000/mL (4,500-11,000/mL). Computed tomography (CT) scanning showed a left necrotic, hemorrhagic renal mass invading the adjacent splenic flexure of the colon. Invasion of the pancreatic tail and left adrenal gland with distal splenic vessels coursing through the mass could also not be excluded (Figure 1). The patient received one dose of aztreonam, vancomycin, metronidazole, and levofloxacin in the emergency department. Piperacillin/Tazobactam was not used, given the patient’s history of penicillin allergy.

After the patient was admitted to the intensive care unit for presumed septic shock, he was treated with an antibiotic regimen of vancomycin, cefepime, and metronidazole. On day two, the patient’s WBC count increased to 49,000/mL (4,500-11,000/mL) and his procalcitonin was elevated to >100ng/mL (<0.25 ng/ml). Blood cultures identified *Clostridium sordellii* as the causative organism, and vancomycin was switched to clindamycin while meropenem was continued.

Over the next week, subsequent blood cultures showed no growth, and the patient’s clinical status stabilized. The antibiotic regimen was switched from meropenem to ertapenem for two weeks upon discharge, followed by oral clindamycin to continue for chronic suppression of the infection until resection of the renal mass. Due to his cancer and dementia progression, his family chose to pursue hospice care soon after his hospital discharge.

## Discussion

This report describes a critically ill patient with back pain who was ultimately diagnosed with a *C. sordellii* infection and successfully treated with a course of antibiotics. The majority of *C. sordellii* cases reported infecting patients during childbirth or gynecologic procedures [1]; however, the patient described in this case presented in the setting of a hemorrhagic necrotic renal mass and its fistulization with the adjacent splenic flexure of the colon. It is suspected that the gastrointestinal tract was the source of this patient’s clostridial infection, which likely reached the bloodstream post-colonic fistulization with the necrotic, hemorrhagic renal mass.

The pathogenicity of *C. sordellii* has been mainly attributed to its hemorrhagic and lethal toxins, which are known to cause local necrosis and edema [1]. These toxins share immunological cross-reactivity with *C. difficile* toxins A and B, being a part of the large family of *Clostridial glucosylating* toxins [11]. These toxins work at the cellular level using similar molecular mechanisms involving glucosylation of Rho and/or Ras GTPAses [11]. When infected, patients may first notice nonspecific symptoms that quickly evolve into massive tissue edema, effusions from the capillary leak, profound leukocytosis, hemoconcentration, refractory hypotension, and tachycardia [1,12]. Typically, on initial presentation, patients infected with *C. sordellii* are already experiencing symptoms of toxic shock, as this patient did, due to its rapidly progressive nature [1,12]. A recent article reported that leukemoid reactions, defined as a WBC count >50,000/ml, were highly suggestive of fatality. This article described 45 cases that had an overall mortality rate of 69%. Of these patients, 80% had a leukemoid reaction, with the majority dying within 2-6 days of infection [1].

As this patient’s history does not follow the typical presentation, this report emphasizes the importance of recognizing the signs and symptoms of this infection and acting quickly due to its high mortality rate. *C. sordellii* must be considered in patients who present in septic shock following a recent surgery or procedure, given the fact that there is no rapid diagnostic test for this infection [1]. This creates a barrier to rapid diagnosis, which can cause a delay in treatment. Upon suspicion of this diagnosis, empiric antibiotic therapy should be started while awaiting blood cultures. While little information exists to support a standard treatment regimen, new and old studies suggest *C. sordellii* is susceptible to beta-lactams, clindamycin, and chloramphenicol and resistant to tetracyclines, aminoglycosides, and sulfonamides [9,10]. Although further investigation is warranted, the use of anti-clostridial toxins as a form of treatment has been suggested and may help guide treatment in such patients [1,2]. 

## Conclusions

We present an interesting case of *Clostridium sordellii* causing toxic shock syndrome posttranslocation through the GI tract. This has not been reported in past literature. The infection is associated with a significant leukemoid reaction. We report successful treatment with clindamycin and meropenem. Clinicians should be aware of this possible presentation with this infectious microorganism. 
